# Efficacy and safety of the 589/1319 nm solid-state dual-wavelength laser combined with topical benzoyl peroxide for inflammatory acne vulgaris: a split-face randomized controlled trial

**DOI:** 10.1007/s00403-025-04146-6

**Published:** 2025-03-26

**Authors:** Suphagan Boonpethkaew, Yanisa Ratanapokasatit, Sonphet Chirasuthat, Penpun Wattanakrai

**Affiliations:** Division of Dermatology, Department of Medicine, Faculty of Medicine, Ramathibodi Hospital, Mahidol University, 270, Rama VI Road, Rajthevi, Bangkok, 10400 Thailand

**Keywords:** Acne vulgaris, Acne treatment, Inflammatory acne, 589/1319 nm dual laser, Non-ablative laser, Benzoyl peroxide

## Abstract

The 589/1319 nm solid-state dual-wavelength (SSDW) laser, which does not require consumable dye, has the potential to target inflammation and sebum production in acne vulgaris pathogenesis. To assess the efficacy and safety of 598/1319 nm SSDW laser as an adjunctive treatment to conventional treatment, 18 patients with bilateral facial acne, with inflammatory papules or pustules, were recruited. Patients were instructed to apply 2.5% benozoyl peroxide (BPO), the drug for inflammatory acne, to their entire face throughout the study. One side of the face was randomly assigned to receive 4 sessions of 589/1319 nm SSDW laser treatments, administered every 2 weeks. After the last laser treatment, 3 monthly follow-ups were scheduled. Inflammatory lesion count (ILC) and acne-related skin parameters, including hemoglobin level, melanin level, skin depression, and skin roughness were measured. Adverse events (AEs) and patients’ satisfaction were assessed. At the 3-month follow-up, the ILC reduced by 46% on the adjunctive laser (BPO + laser) side (*p* = 0.0080), compared with a 29% reduction on the BPO monotherapy side (*p* = 0.1875). On the adjunctive laser side, the change in ILC positively correlated with the change in melanin level (*r* = 0.51, *p* = 0.0301) and showed a trend towards a positive correlation with the change in depression volume (*r* = 0.45, *p* = 0.0606) and roughness level (*r* = 0.42, *p* = 0.0806). The patients reported a pain score of 3.4 ± 2.3 on scale of 10. No serious AEs occurred. Patients’ satisfaction scores were higher with the adjunctive laser therapy, although this was not statistically significant (*p* = 0.2758). In conclusion, the 589/1319 nm SSDW laser provided a synergistic effect as an adjunctive treatment to BPO in inflammatory acne in terms of reducing ILC and improving post inflammatory hyperpigmentation without causing discomfort or downtime.

**Supplementary Information:**

The online version contains supplementary material available at 10.1007/s00403-025-04146-6.

## Introduction

Acne vulgaris is clinically characterized by the presence of comedones, inflammatory papules/pustules, or nodules in the follicular areas, especially on the face, thus causing a profound impact on both the physical and psychological well-being of affected individuals [1, 2]. Its pathogenesis is driven by 4 major underlying mechanisms: increased sebum production, hyperkeratinization of the pilosebaceous unit, dysbiosis of the microbiome, and inflammation [3, 4].

Numerous therapeutic modalities, including topical medications, oral antibiotics, and systemic retinoids, have long been available, and guidelines for good clinical practice have been established [5, 6]. In addition to standard topical and systemic medications, lasers have emerged as promising tools for reducing inflammation [e.g., 585–595 nm pulse dye laser (PDL), long-pulsed 1064 nm neodymium-doped yttrium aluminum garnet (Nd-YAG)] and controlling sebum production [e.g., 1064 nm Nd-YAG, 1450 nm diode laser (DL), 1565 nm non-ablative fractional laser (NAFL)] in the pathogenesis [7]. Additionally, laser combinations such as PDL combined with Nd-YAG laser, DL, or 1565 nm NAFL to address multiple underlying mechanisms of acne vulgaris and reduce acne complications have shown favorable outcomes [8–12]. However, in clinical practice, some patients experienced discomfort from pain and undesirable side effects from the lasers [7].

The 589/1319 nm solid-state dual wavelength (SSDW) laser is a non-ablative laser, offering patients a downtime-free experience. The 589 nm wavelength produces a higher energy spike of PDL wavelength that directly targets the chromophores hemoglobin without damaging surrounding tissues [13]. Additionally, the near-infrared (NIR) 1319 nm wavelength can be absorbed by water and generates heat, which reduces sebaceous gland activity [14] and stimulates neocollagenesis [15]. Thus, the potential for synergy between these two wavelengths to target the underlying mechanisms of inflammatory acne and prevent acne complications such as acne scarring.

Therefore, this study investigated the efficacy and safety of the 589/1319 nm SSDW laser as an adjunctive treatment to standard topical benzoyl peroxide (BPO) in inflammatory acne.

## Materials and methods

### Research design

This study was a single-blinded, split-face, randomized controlled trial conducted at Ramathibodi Hospital, Thailand. We aimed to compare the efficacy of the 589/1319 nm SSDW laser adjunctive to topical 2.5% BPO versus topical 2.5% BPO monotherapy for the treatment of inflammatory acne. The trial was registered at www.thaiclinicaltrials.org as TCTR20221003006.

### Subject recruitments

Eighteen healthy subjects aged 18–50 years old with bilateral facial acne vulgaris and at least 2 inflammatory papules or pustules on each facial side were enrolled. The washout period prior to participation for systemic treatments (isotretinoin and doxycycline) was 3 months and 1 month for topical treatments (BPO, clindamycin, metronidazole, adapalene, tretinoin, glycolic acid, and/or salicylic acid). Subjects who were pregnant, lactating, sensitive to light, or had a history of other laser therapies for facial acne treatment were excluded. The study was conducted according to the Declaration of Helsinki and was approved by the Mahidol University Institutional Review Board for Ethics in Human Research (COA. MURA2022/556). Informed consent and photo consent were obtained from all subjects before they were included in the study.

### Treatment protocol

The patients were instructed to apply 2.5% BPO (Benzac AC^®^ 2.5, Galderma, Montdésir, France) to the entire face once daily for 30 min before rinsing it off throughout the study period (18 weeks). Either left or right facial sides were randomized (block randomization) to receive the 589/1319 nm SSDW laser treatment (ADVATx^®^, Advalight, Ballerup, Denmark) every 2 weeks for a total of 4 sessions. The laser parameters were as follows: beginning with the 589 nm wavelength at a fluence of 40 J/cm^2^ to achieve a total energy of 650 J by gliding technique, followed by the 1319 nm wavelength at a fluence of 60 J/cm^2^ to achieve a total energy of 1300 J by gliding technique for the entire half of the randomized facial side. Laser pulses were delivered by a 10 × 10 mm square scanner, with a repetition mode of 0.25 s and a high-density filling factor. Finally, only the inflammatory lesions on the randomized facial side were treated with the 589 nm wavelength at a fluence of 12 J/cm^2^ (1 shot/lesion) using a 5 × 5 mm square scanner, with a repetition mode of 0.25 s and a medium-density filling factor. All patients were instructed to apply moisturizer (Cetaphil^®^ moisturizing cream, Galderma, Montdésir, France) twice daily and sunscreen with SPF 50^+^/UVA 28 (Cetaphil^®^ UVA/UVB defense, Galderma, Montdésir, France) at least once in the morning. The follow-up sessions were scheduled monthly for a total of 3 months after the last laser treatment.

### Outcome assessment

#### Subjective assessments

##### Physician assessment

The investigator’s global assessment (IGA) of acne severity grade [16, 17] was modified to assess the half-face acne severity from patients’ photographs taken with Visia CR^®^ camera (Fairfield, NJ, USA) by 2 independent dermatologists with a blinding method (Table 1).

**Table 1:** Modified investigator’s global assessment of acne severity grade

Grade		Description
0	Clear	No inflammatory lesions and no comedones ± Residual hyperpigmentation or erythema
1	Almost clear	Few small papules and few scattered comedones
2	Mild	Easily recognizable; < 50% of the half face Some papules/pustules and some comedones
3	Moderate	> 50% of the half face Many papules/pustules and many comedones ± One nodule
4	Severe	Entire the half face Numerous papules and pustules and comedones Few nodules and cysts

##### Patient assessment

A visual analog scale (VAS), ranging from 0 to 10, was used to assess the pain score of laser treatments (0 = no pain, 5 = moderate pain, and 10 = intense pain) and the overall satisfaction score (0 = no satisfaction, 5 = moderate satisfaction, and 10 = high satisfaction).

#### Objective assessment

Inflammatory acne lesions were counted (ILC) from the patients’ photographs by 2 blinded dermatologists. Antera 3D^®^ camera (Miravex Co., Ltd, Dublin, Ireland) was utilized to measure acne-related skin parameters, including melanin level, hemoglobin (Hb) level, depression volume and roughness level. Adverse events (AEs) were monitored throughout the study period.

### Statistical analysis

Descriptive data was presented as number (percentage), mean [standard deviation (SD)], and median (min–max), as appropriate. The repeated-measures ANOVA and mixed-effects model were used to compare the statistical differences in the means of multiple comparisons. The Chi-squared test was used to analyze the statistical differences in categorical data. GraphPad Prism version 10.2.2 was utilized for all statistical analyses and graph presentations. Statistical significance was set at *p* < 0.05.

## Results

### Eighteen patients completed 4 laser sessions and 3-month follow-ups

A total of 18 patients with mild to moderate acne vulgaris with inflammatory lesions participated in the study. The demographic data of the subjects is shown in Table 2.

**Table 2:** Patient demographic data

Patient with acne vulgaris, n (%)	18 (100)
Female	15 (83.33)
Male	3 (16.67)
Age (year)	
Mean (SD)	26.94 (6.68)
Median (range)	27 (18–41)
Fitzpatrick skin type, n (%)	
III	5 (27.78)
IV	9 (50)
V	4 (22.22)
Acne severity grade (IGA), n (%)	
Moderate	10 (55.56)
Mild	8 (44.44)
Chronicity of acne vulgaris in years, median (range)	3 (1–20)

IGA, investigator’s global assessment

### The 589/1319 nm SSDW laser adjunctive to 2.5% BPO significantly reduced ILC 46% at 3 months

**Fig. 1:**
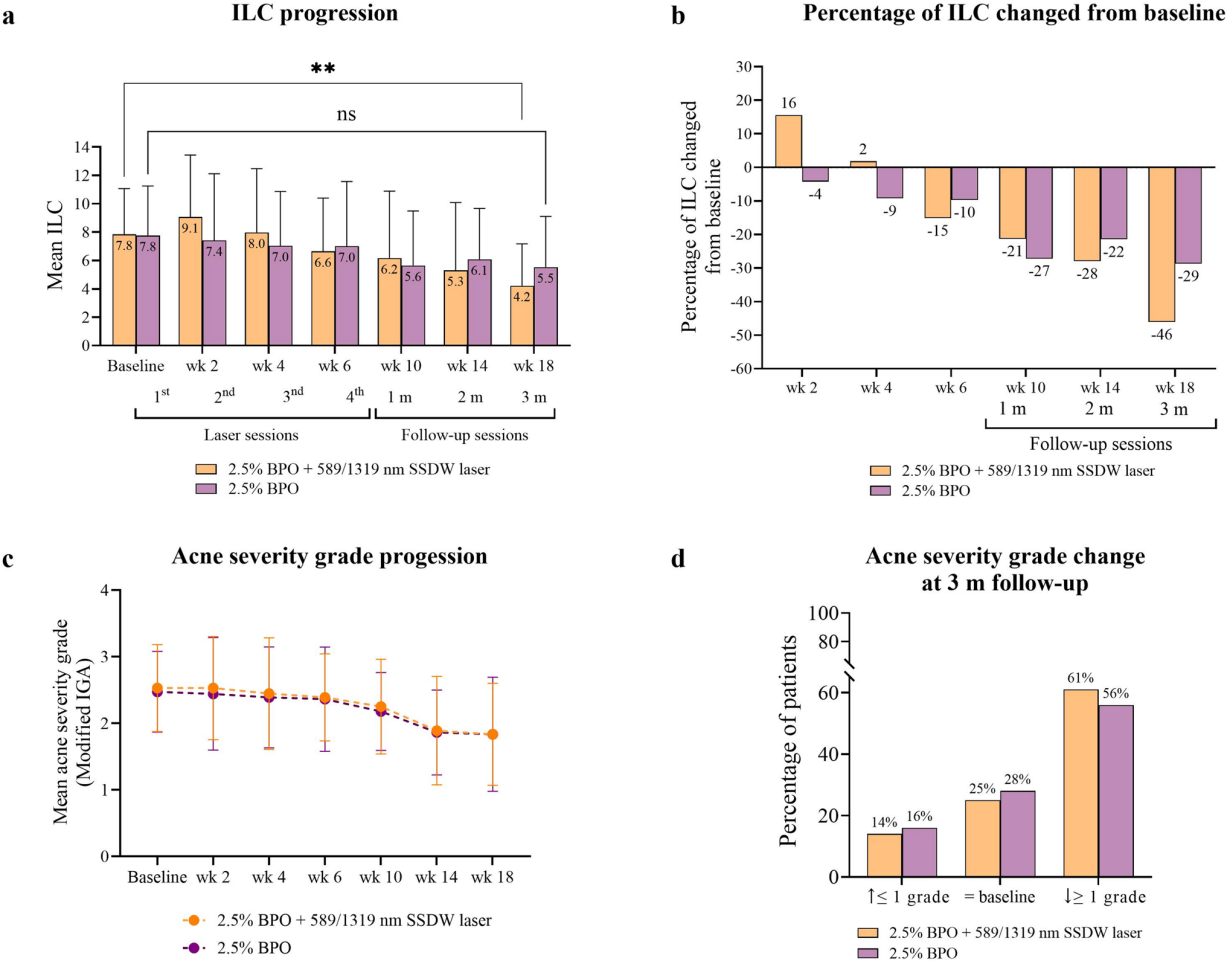
ILC and acne severity grade. **a** Mean of ILC. **b** Percentage of ILC changed from baseline. **c** Mean of acne severity grade. **d** Acne severity grade changed from baseline at the 3-month follow-up. n = 18 each group, **p < 0.01. BPO, benzoyl peroxide; ILC, inflammatory lesion count; m, month; nm, nanometer; SSDW, solid-state dual wavelength; wk, week

Three months after completing 4 laser treatments, the ILC reduced by 46% from baseline on the BPO + laser therapy (*p* = 0.0080) compared to a 29% reduction on the BPO monotherapy side (*p* = 0.1875) (Fig. 1a, b). However, the ILC was not statistically different between groups (*p* = 0.9200). The acne severity grade (modified IGA) progressively decreased by both groups throughout the study period (between-group comparison, *p* = 0.7631) (Fig. 1c). Additionally, at the 3-month follow-up, 61% of the adjunctive laser sides achieved at least 1-grade reduction of the modified IGA, compared to 56% of the BPO monotherapy side (*p* = 0.8127) (Fig. 1d).

### ILC change positively correlated with melanin level change on the adjunctive laser therapy

**Fig. 2:**
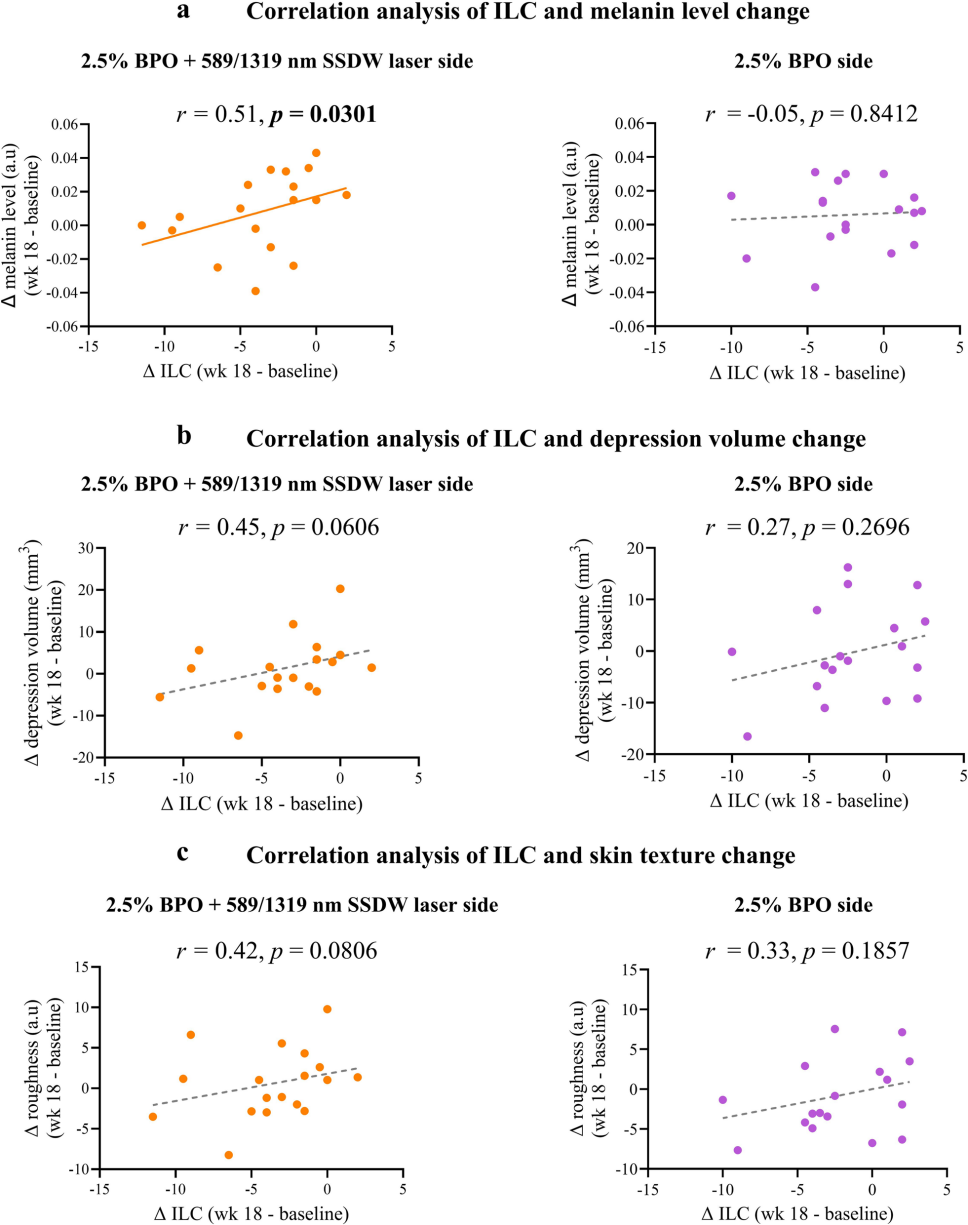
Correlation between the change in ILC and the change in acne-related skin parameter. The correlation analysis of the change in ILC with the change in **a** melanin level, **b** depression volume, and **c** roughness level on both the adjunctive laser side and the BPO monotherapy side was performed using Spearman's correlation coefficients (*r*). BPO, benzoyl peroxide; ILC, inflammatory lesion count; nm, nanometer; SSDW, solid-state dual wavelength; wk, week

Among the average acne-related skin parameters (Figure S1), the Hb level decreased by 7.82% (*p* = 0.9667) on the adjunctive laser side at the 3-month follow-up, compared to 5.59% (*p* = 0.3836) on the BPO monotherapy side (between-group comparison, *p* = 0.6607). Although the average melanin level, depression volume, and roughness level remained stable throughout the study period, the change in these parameters of each patient correlated with the change in ILC. Specifically, the change in ILC had a significant positive correlation with melanin level change on the adjunctive laser side (Fig. 2a). Additionally, the change in ILC also displayed a trend towards a positive correlation with the change in depression volume and skin texture (roughness level) on the adjunctive laser side (Fig. 2b, c).

### Patients reported minor AEs and higher satisfaction score with the adjunctive laser therapy

**Fig. 3:**
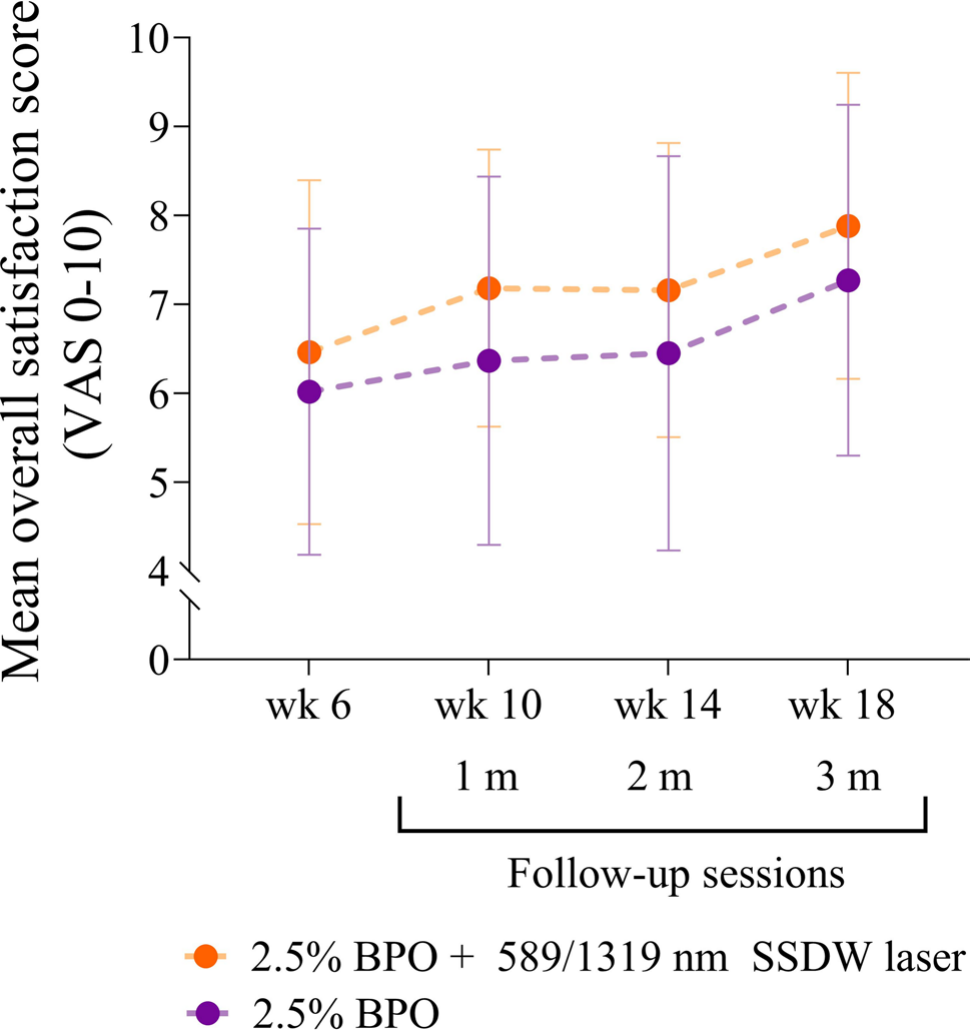
Pain score of 589/1319 nm SSDW laser and patient satisfaction. Mean overall satisfaction score (n = 18 each group). BPO, benzoyl peroxide; ILC, inflammatory lesion count; m, month; nm, nanometer; SSDW, solid-state dual wavelength; VAS, visual analog scale; wk, week

The patients reported an average pain score of 3.4 ± 2.3 on scale of 10 for the 589/1319 nm SSDW laser treatment. No serious or unpredictable AEs were reported or observed throughout the study period. However, 16.67% (3/18) of the patients experienced mild dryness, erythema, and peeling on both sides of the face; however, these symptoms spontaneously resolved within 2 weeks. Although no patient reported acne flare-ups, the ILC increased by 16% in the 2nd week after the 1st laser treatment but subsided in the 4th week after 2 laser sessions (Fig. 1b). The patients reported higher satisfaction scores with the adjunctive laser therapy compared to the BPO monotherapy, although this did not reach statistical significance (*p* = 0.2758) (Fig. 3).

## Discussion

Acne vulgaris poses a significant challenge in treatment, especially the inflammatory lesions. Early treatment and suitable intervention strategies to reduce inflammation are essential to prevent complications such as acne erythema, post-inflammatory hyperpigmentation (PIH), and acne scar. In this study, the efficacy and safety of the 589/1319 nm SSDW laser adjunctive to topical 2.5% BPO for inflammatory acne treatment was studied.

**Fig. 4:**
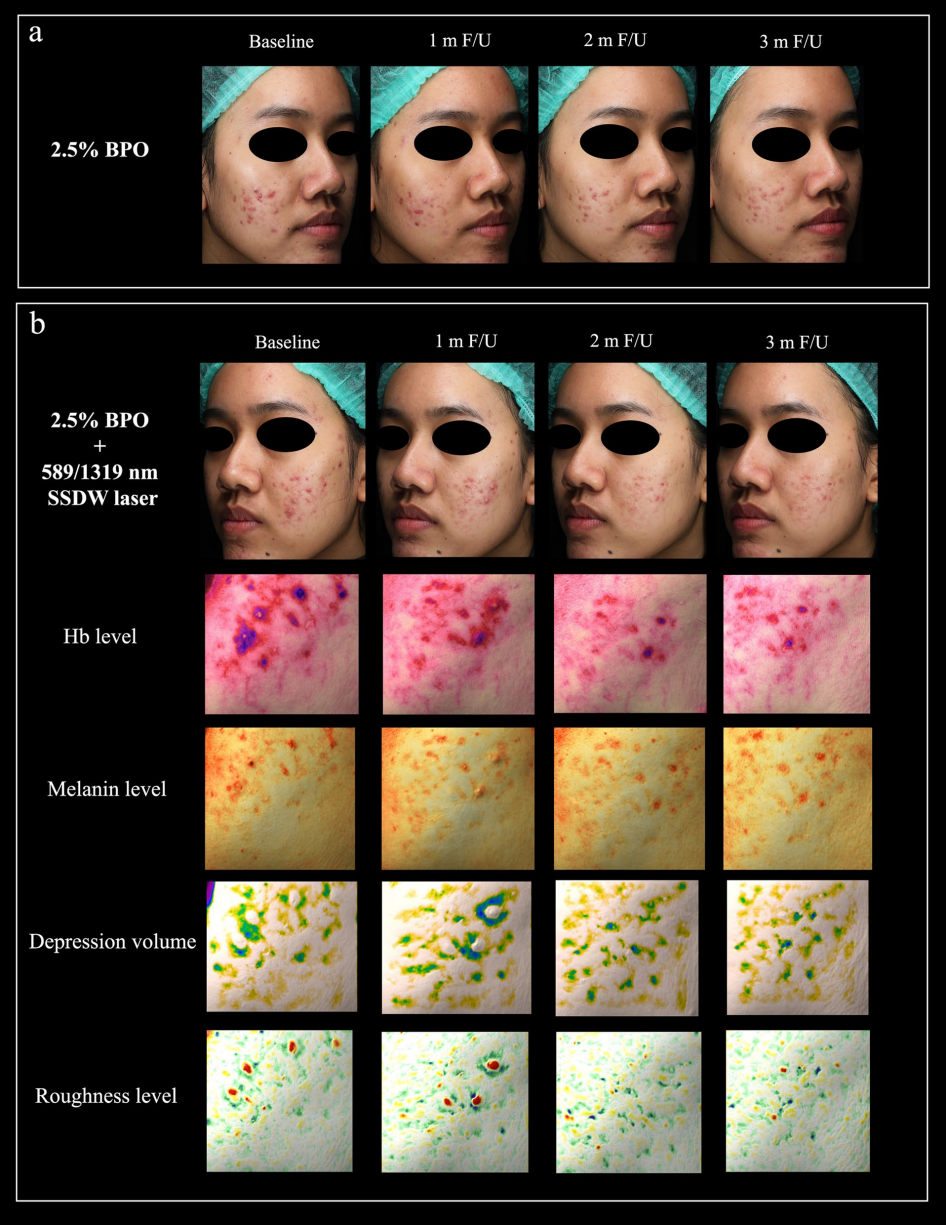
Clinical and acne-related skin parameter photographs. **a** The clinical improvement of BPO monotherapy side. **b** The clinical improvement and acne-related skin parameters of adjunctive laser side. BPO, benzoyl peroxide; F/U, follow up; m, month; nm, nanometer; SSDW, solid-state dual wavelength

The results demonstrated that the 589/1319 nm SSDW laser adjunctive to BPO could significantly reduce 46% of the ILC 3 months after completing 4 laser sessions. In comparison, the BPO monotherapy reduced 29% of ILC. This indicated that the laser offered an additional 17% reduction in the ILC compared to the BPO monotherapy. The photograph of one of the patients who achieved favorable results in terms of ILC reduction and acne-related skin parameters is shown in Fig. 4. Our findings were consistent with a recent report from Lim [18] which used the 589/1319 nm SSDW laser (2–3 week interval, 5 sessions) as an adjunctive to topical retinoids (0.1% Adapalene or 0.025% Tretinoin) in 40 patients with moderate to severe acne vulgaris. Results indicated that this laser therapy + topical retinoids was effective and safe, with 73.3% of the patients experiencing a ≥ 75% reduction of ILC at the 3-month follow-up. However, determining the additional effects of the laser to topical retinoids was challenging due to the lack of a control group. Another study conducted by Kang et al. [9] utilized a split-face methodology in 9 subjects with moderate to severe inflammatory acne and reported that after 4 sessions of 589/1319 nm SSDW laser treatments, the ILC were decreased by 23%, with 85.7% of patients showing improvement, in contrast to an 11% ILC increase on the untreated side. In comparison to our study results, the facial areas treated with four 589/1319 nm SSDW laser sessions in conjunction with topical BPO showed a 21% reduction in ILC at 1-month follow-up, which further increased to 28% and 46% at the 2-month and 3-month follow-ups, respectively. These findings confirm that the 589/1319 nm SSDW laser is effective in reducing ILC both as a monotherapy and, more notably, when used as an adjunct to topical BPO.

The potential mechanisms of this 589/1319 nm dual wavelength laser are discussed. When specifically reviewing monowavelength lasers for the treatment of acne vulgaris, the focus was on two wavelength ranges: visible wavelengths between 585–595 nm and NIR wavelengths between 800–2400 nm. The meta-analysis was revealed that 585–595 nm PDL with long pulse width (≥ 10 ms) and multiple sessions (≥ 4 sessions) could reduce acne severity scores as monotherapy [19]. The PDL wavelength targets acne vasculature by absorption of Hb and increases transforming growth factor-β, thereby reducing inflammation [7, 20]. Correspondingly, our study confirmed that the 589/1319 nm SSDW laser in adjunct to BPO provided additional benefit in reducing the Hb level measured in the skin (Figs. 4, S1a). Specifically, adjunctive laser therapy resulted in a 7.82% reduction in Hb level at the 3-month follow-up, while BPO monotherapy resulted in a 5.59% reduction. Although this change did not reach statistical significance, it may indicate that superficial inflammatory microvasculature was destroyed.

Focusing on NIR wavelength lasers, such as the 1064 nm Nd:YAG laser, the 1320 nm Nd:YAG laser, the 1450 nm DL, the 1540 nm erbium laser, and the 1726 nm DL, studies have demonstrated favorable results in the treatment of acne vulgaris by producing selective photothermolysis that damages the sebaceous glands, subsequently decreasing sebum production and reducing *Cutibacterium acnes* density [7, 21–26]. Among NIR wavelengths, the peak absorption ratio of sebum over water occurs at 1726 nm [22, 27]. Studies using the 1726 nm DL as monotherapy (7-week interval, 1–3 sessions) showed a reduction in the ILC by 56% at the 3-month follow-up. When the 1726 nm DL laser treatment interval was shortened (3-week interval, 3 sessions), 79.8% of patients achieved a ≥ 50% reduction in the ILC at the 3-month follow-up [22]. Therefore, combining two ranges of wavelength lasers (585–595 nm and NIR) could target multiple aspects of acne vulgaris pathogenesis, including inflammation, sebum hyperproduction, and *C. acnes* infection. Before the invention of the 589/1319 nm SSDW laser, a study combining treatments with two laser devices; the 595 nm PDL with the 1450 nm DL, as adjunctive treatment to patients’ acne regimen, including topical medications (not limited to BPO) and/or oral antibiotics, demonstrated a 84% ILC reduction 3 months after a total of 3 sessions (4–6-week intervals) [10].

Interestingly, our results revealed additional benefits of using the 589/1319 SSDW laser for acne vulgaris treatment. The change in ILC exhibited a significant positive correlation with the change in melanin level on the adjunctive laser therapy side, but not on the BPO monotherapy side. This could be interpreted that the patient who experienced a greater ILC reduction also experienced a greater melanin level reduction at the 3-month follow-up. This could be explained by greater reduction of inflammatory lesions which should lead to less PIH or could be due to the melanin chromophore which can also absorb the 585–595 nm wavelength [28]. Meanwhile, the mechanisms of action of BPO include bactericidal effects on *C. acnes*, anti-inflammatory properties, and comedolysis without directly affecting the pigmentation process [29]. Moreover, the change in ILC exhibited a trend towards a positive correlation with the change in depression volume and roughness level. Our results were consistent with a study by Gold et al., which used the 589/1319 nm SSDW laser to treat acne scars in 10 patients and achieved a 40% reduction in the échelle d’évaluation clinique des cicatrices d’acné (ECCA) score at one month after 4 laser sessions [30]. This can be explained by the hypothesis that 1319 nm wavelength could be absorbed by water, generating heat to stimulate neocollagenesis and collagen remodeling. This was supported by a study conducted by El-Domyati et al. [15] which observed the synthesis of collagen types I, III, and VII, as well as tropoelastin (new elastin) after 1320 nm Nd:YAG laser treatment. In addition to 1319 nm, the PDL wavelengths could also induce the collagen remodeling processes [31, 32]. Therefore, it was clear that the patient whose inflammation subsided might experience accelerated remodeling due to the synergy of 589 nm and 1319 nm wavelength. So, the potential mechanism of the 589/1319 nm SSDW laser in treating acne and its complications is to reduce inflammation by the 589 nm laser, decrease sebum production by the 1319 nm laser, minimize PIH by the 589 nm laser, and stimulate collagenogenesis and tissue remodeling by both the 589 nm and 1319 nm lasers.

In terms of AEs, the patients reported the average pain score of 3.4 out of 10 for the 589/1319 nm SSDW laser, which aligned closely with the report from Singapore (~ 3.7/10) [18]. However, when compared to the average pain score from other previous studies treating acne vulgaris with 595 nm PDL (~ 5.3/10) and 1726 nm DL (~ 4.9/10) [21, 33], the pain score of 589/1319 nm SSDW laser was relatively lower. Three patients reported mild dryness, erythema, and peeling on both sides of the face. However, these symptoms resolved without treatment within 2 weeks. These symptoms could be the predictable AEs of BPO [34]. Additionally, physicians observed a transient 16% increase in ILC with the adjunctive therapy, which spontaneously decreased from the 4th week after the initial treatment onward. We suspected that the 1319 nm wavelength may induce acne flare-up, as observed with other NIR wavelength lasers like 1550 nm and 1726 nm [21, 22, 35]. The flare-up was suspected to be caused by follicular destruction triggering follicular inflammation, with a lesser degree observed with lower energy beam [35].

While our study provides an option for inflammatory acne treatment, it has limitations: a low number of patients, a single ethnicity represented, the need for further study on optimal laser parameters, and the consideration of adjunctive use with other acne treatment regimens.

In conclusion, the 589/1319 nm SSDW laser was proven to provide a synergistic effect, with minimal discomfort and downtime, as an adjunctive treatment to BPO for inflammatory acne vulgaris. In addition to decreasing *C. acne*, reducing inflammation, and enhancing comedolysis, the adjunctive laser treatment, which resulted in higher ILC reduction and satisfaction, may simultaneously help improve PIH and stimulate the collagen remodeling process.

## Supplementary Information

Below is the link to the electronic supplementary material.

Supplementary file1 (DOCX 722 KB)

## Data Availability

The data that support the findings of this study are available from the corresponding author upon reasonable request. No datasets were generated or analysed during the current study.
