# Supplementary material for: Efficacy and safety of the 589/1319 nm solid-state dual-wavelength laser combined with topical benzoyl peroxide for inflammatory acne vulgaris: a split-face randomized controlled trial
Source: Arch Dermatol Res. 2025 Mar 26;317(1):635. doi: 10.1007/s00403-025-04146-6 (PMC11947063; doi:10.1007/s00403-025-04146-6)

**Appendices**

**Figure S1** Acne-related skin parameters

The average level of (a) hemoglobin, (b) melanin, (c) depression volume, and (d) roughness level. The graphs depicted the mean and standard deviation. The differences between groups were analyzed using repeated-measures ANOVA. n = 18 in each group.

*a.u., arbitrary unit; BPO, benzoyl peroxide; Hb, hemoglobin; nm, nanometer; SSDW, solid-state dual wavelength; wk, week.*


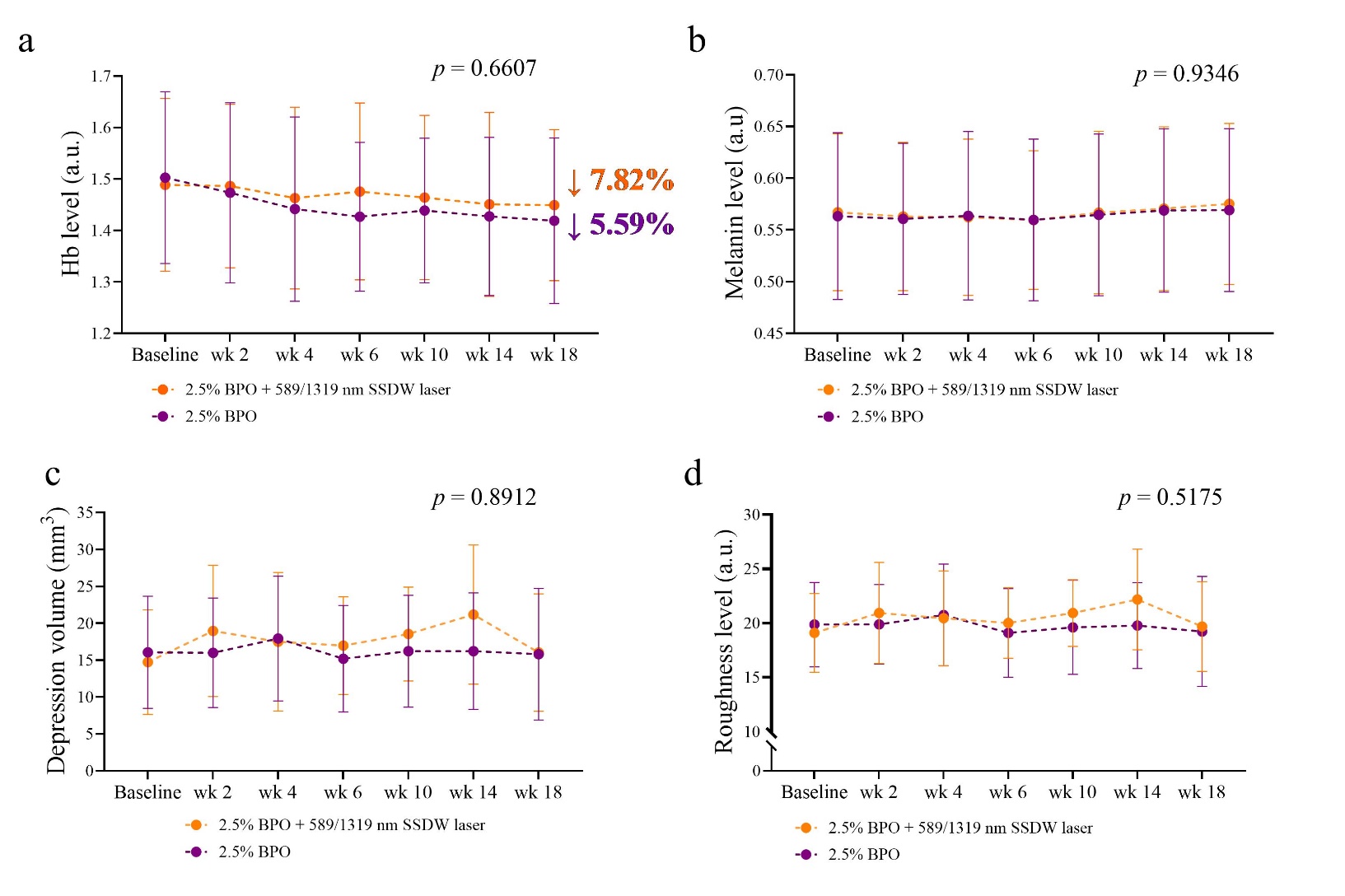

Supplement: Supplementary file 1 — Supplementary file1 (DOCX 722 KB) [file 403_2025_4146_MOESM1_ESM.docx]
